# Biome: evolution of a crucial ecological and biogeographical concept

**DOI:** 10.1111/nph.15609

**Published:** 2018-12-26

**Authors:** Ladislav Mucina

**Affiliations:** ^1^ Iluka Chair in Vegetation Science and Biogeography School of Biological Sciences The University of Western Australia 35 Stirling Hwy, Crawley Perth WA 6009 Australia; ^2^ Department of Geography & Environmental Studies Stellenbosch University Private Bag X1, Matieland Stellenbosch 7602 South Africa

**Keywords:** azonal biomes, biogeography, biome modelling, climate, evolution of biome, genomic tools, plant functional types, vegetation zonality

## Abstract

A biome is a key community ecological and biogeographical concept and, as such, has profited from the overall progress of community ecology, punctuated by two major innovations: shifting the focus from pure pattern description to understanding functionality, and changing the approach from observational to explanatory and, most importantly, from descriptive to predictive. The functional focus enabled development of mechanistic and function‐focused predictive and retrodictive modelling; it also shaped the current understanding of the concept of a biome as a dynamic biological entity having many aspects, with deep roots in the evolutionary past, and which is undergoing change. The evolution of the biome concept was punctuated by three synthetic steps: the first synthesis formulated a solid body of theory explaining the ecological and biogeographical meaning of zonality and collated our knowledge on drivers of vegetation patterns at large spatial scales; the second translated this knowledge into effective mechanistic modelling tools, developing further the link between ecosystem functionality and biogeography; and the third (still in progress) is seeking common ground between large‐scale ecological and biogeographic phenomena, using macroecology and macroevolutionary research tools.

‘… concepts tend to evolve in time, and so confusion and pointless controversy can be avoided by looking at their lineages…’(Jackson, 2009)

## Ante prima

At least on land, plants form and dominate biotic communities. These communities are structured along spatial and temporal scales. At very large scales, we call these biotic communities and their environments form biomes.

This review is about ‘biomes’ – a term that was coined almost exactly 100 yr ago, and which, ever since, has made many appearances, and experienced an interesting and illustrious evolution. There appears to be a consensus in ecology and biogeography that a biome is a useful tool, as it denotes a biotic community finding its expression at large geographic scales, shaped by climatic factors, and perhaps better characterized by physiognomy and functional aspects, rather than by species or life‐form composition. Biomes are frequently used as tools to provide large‐scale (regional to global) backgrounds in a range of ecological and biogeographical studies. Among such studies are those addressing global biodiversity conservation efforts (e.g. Olson *et al*., 2001), land‐use dynamics (e.g. Loveland *et al*., 2000; Ellis & Ramankutty, 2008; Bodart *et al*., 2013), fluxes of matter and energy (e.g. Jackson *et al*., 1996; Turner *et al*., 2006; Yi *et al*., 2010; Stocker *et al*., 2018), and climate change (e.g. Williams *et al*., 2007; Gonzalez *et al*., 2010; Li & Zhang, 2017). Using a biome as a tool in this way requires us to understand the patterns and drivers of life at large spatial (and temporal) scales. Formulating a scientifically sound biome scheme to assist in tackling problems of planetary change should be one of our priorities. This aim, however, is not achievable if we do not appreciate the origins, evolution and current understanding of biomes as a concept. This review, by focusing on terrestrial biomes and leaving aside marine biomes, aims to provide guidance in this matter and to identify those research needs that should be the focus of biome ecologists.

### The days of the grandfathers

There are four concepts that are considered precursors to the biome: association, formation, biocenose and life zone.

Association is a famous term coined by Alexander von Humboldt who was a believer in ‘social organised plant life’ (von Humboldt & Bonpland, 1805: 7; Jackson, 2009 edition: page 67; see Nicolson, 2013) – an idea that gave birth to phytosociology (Braun‐Blanquet, 1964), which is today called vegetation science. Association, a type of ecological community with a predictable species composition and consistent physiognomy which occurs in a habitat type, is a key term in vegetation science.

Grisebach (1838) introduced a new term that would become very influential in vegetation science and biogeography – ‘formation’. Warming (1909) used the terms ‘community’ and ‘formation’ as synonyms (Egerton, 2017), which is essentially correct if we consider ‘formation’ as a large‐scale plant community. Schimper (1898) shaped the concept of formation decisively by demonstrating its usefulness on a global scale.

Möbius (1877) wrote about a ‘community of living beings’ and a ‘collection of species’ when he coined his term ‘biocenose’ (or ‘biocoenosis’), which gave birth to concepts such as phytocoenosis and zoocoenosis.

The fourth term completing the mosaic of conceptual precursors to the term biome is Merriam's (1892, 1894) ‘life zone’, which demonstrates a spatially explicit relationship between distribution of biota and climate.

These four terms set the scene for the almost serendipitous advent of the term ‘biome’, although conceptually the concept itself had already been heralded by Schimper (1898).

### Advent and early development of the biome concept

The term biome was born in 1916 in the opening address at the first meeting of the Ecological Society of America, given by Frederick Clements (1916b). In 1917, an abstract of this talk was published in the *Journal of Ecology*. Here Clements introduced his ‘biome’ as a synonym to ‘biotic community’. Only later did Shelford & Olson (1935) clarify that this is a biotic community of a special kind – pertinent to large geographic scales, representing a climax (Clements, 1916a) and ‘a community of formation rank in the largest sense of the term’. Tansley (1935) interpreted biome as ‘the whole complex of organisms inhabiting a given region’. Finally, Clements & Shelford (1939) established the link between the theory of the vegetation climax and the biome. Although the notion of a ‘climax’ has always been contentious, because of its clear stable‐equilibrium flavour (Pickett, 1980) and its far‐fetched analogy (complex organism, social organism) with the organism, it was still inspirational in defining zonobiomes (Walter, 1942).

The rebranding of the Clements’ (1917) biome as a large‐scale community was completed in Shelford (1945), who understood the biome as the largest‐scale unit of his system of North American biomes. Although Clements & Shelford (1939) did not acknowledge the spatial aspect of the biome explicitly, the concept is owned equally today by geography and ecology (see, for instance, Hanks, 2011).

## Drivers of community patterns at large spatial scales

### Role of scales and drivers

Hierarchy theory (e.g. Allen & Starr, 1982; Kolasa & Pickett, 1989) teaches us that ecological factors operate at various embedded spatial and temporal scales – the functionality is hierarchically ordered. At each spatial and temporal scale, a different set of variables is driving the formation of community patterns; sets of assembly rules were defined that were unique to different spatial scales and timescales. The hierarchically scaled factors produce complex hierarchical patterns known as communities, groups of communities, and, of course, biomes (following an axis of increasing complexity).

Of all environmental drivers, macroclimate is considered the most important structuring/driving factor of biota at large scales. Biotic interactions aside, soils and fine‐scale water availability govern the finer scales. Both create a series of filters, selecting for the best‐suited traits and trait syndromes that determine vegetation physiognomy. Macroclimate, soil, water and, finally, disturbance are the major ingredients from which biomes – in all their appearance, complexity and functioning – are ‘cooked’.

### Climate rules the world

Global climatic zonation is one of the oldest findings known to science. Notions of ‘frigid’ (cold), ‘temperate’ and ‘torrid’ (hot) zone have been ascribed (e.g. Coxon & McKirahan, 2009) to the Greek philosopher Parmenides (see Table 1). Shugart & Woodward (2011) noted that relationships between climate and vegetation are among the earliest ecological observations, dating back to the third century BC, by Theophrastos.

**Table 1:** Milestones of the development of the biome ecology

Year	Milestones	Primary source
Fifth century BC	First notion of the global climatic zonation	Parmenides (fifth century BC)
1792	Recognition of the influence of climate on global distribution of vegetation	Willdenow (1792)
1805	Birth of geography (and biogeography); formalization of elevational zonation	von Humboldt & Bonpland (1805)
1838	Definition of the concept of formation (in vegetation and climate context)	Grisebach (1838)
1894	Life zone defined for the first time: a large‐scale precursor of a biome	Merriam (1894)
1898	First attempt to explain global vegetation patterning (temperature and water); roots of the biome concept	Schimper (1898)
1899	Formulation of a global zonation of soils	Dokuchaev (1899)
1900	First global climatic classification	Köppen (1900)
1905	First comprehensive life‐form system	Raunkiær (1905)
1917	First mention of the term ‘biome’ as a synonym to biotic community	Clements (1917)
1933	New global classification of climatic zones	Thornthwaite (1933)
1935	Introduction of the concept of ecosystem	Tansley (1935)
1939	Recognition of biome as a large‐scale concept	Clements & Shelford (1939)
1943	Concept of biotic province	Dice (1943)
1945	Sheldord's meeting (proceedings published in *Wilson Bulletin*)	Shelford (1945), Odum (1945)
1945	First record of the term ‘evolution of biome’	Odum (1945)
1947	Climatic redefinition of life zone	Holdridge (1947)
1954	The first synthesis: zonality and azonality of biomes formalized	Walter (1954b)
1964	Altitudinal zonality type	Lavrenko (1964)
1964	First global functional model of vegetation cover based on net primary productivity	Lieth (1964)
1968	Defining (evolution of) biome in functional‐dynamic terms	Valentine (1968)
1970	Redefinition of biome in climatic space	Whittaker (1970)
1976	Introduction of the concept of ecoregion	Bailey (1976)
1981	The second synthesis: first predictive models developed	Box (1981a,b)
1990	Multiple stable states of biomes	Dublin *et al*. (1990)
1992	BIOME: first equilibrium‐coupled biome model	Prentice *et al*. (1992)
1995	Ecozone	Schultz (1995)
1996	Bioclimatic zones of Europe	Rivas‐Martínez (1996)
2001	Biome defined as a lump sum of ecoregions	Olson *et al*. (2001)
2001	FAO ecological zone	FAO (2001)
2001	Ecosystem functional type: the first functional biome concept	Paruelo *et al*. (2001)
2006	Zonobiome redefined: large‐scale disturbance explicitly part of the concept	Rutherford *et al*. (2006)
2008	Anthropogenic biomes (anthromes)	Ellis & Ramankutty (2008)
2013	Next generation of functional biome models	Scheiter *et al*. (2013)
2013	Biome map of Europe based on vegetation map	Mucina (2013)
2016	The third synthesis: linking environmental templates, traits and evolution	Moncrieff *et al*. (2016)
2016	Special issue of *South African Journal of Botany* on biomes	*South African Journal of Botany*
2016	A new type of functional biome	Higgins *et al*. (2016)

**Figure 1:**
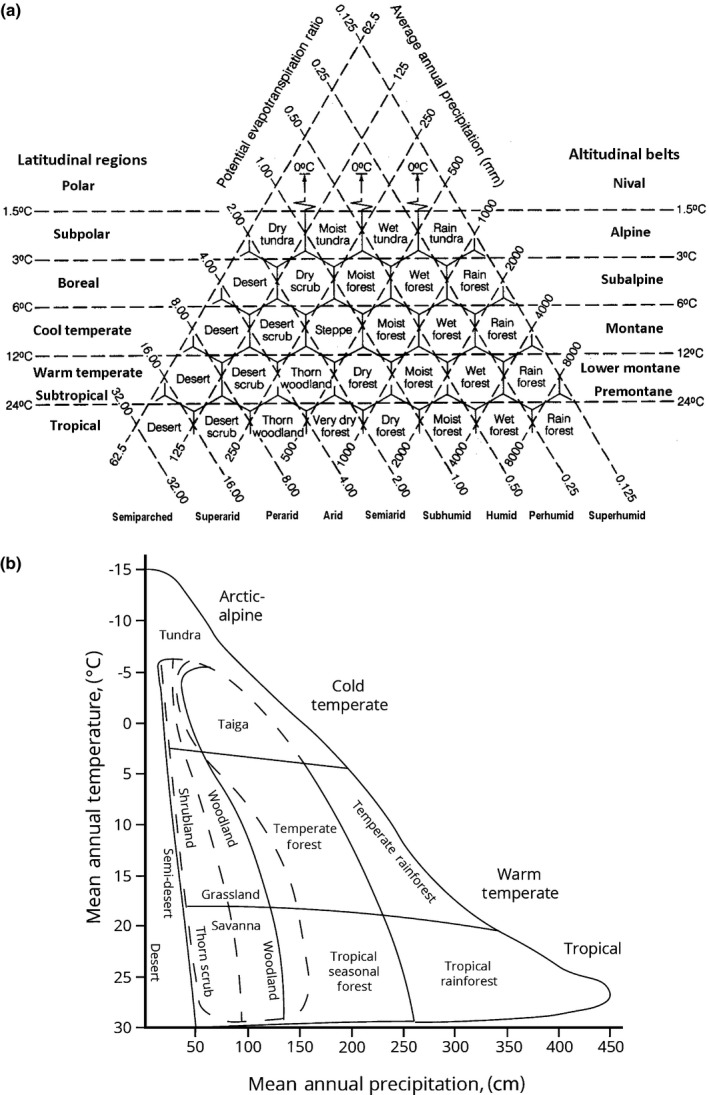
Two very popular biome schemes based on bioclimatic approach: (a) Holdridge's scheme (reproduced from Archibold, 1995; after Holdridge, 1947); (b) Whittaker's scheme (after Whittaker, 1970). Reproduced with permission from Elsevier Ltd (a) and Prentice Hall/Pearson Higher Education (b).

Climate is implicated as the major driver of vegetation patterning, shaping the physiognomy of vegetation cover (e.g. Grisebach, 1838; Schimper, 1898; Clements & Shelford, 1939; Walter, 1954b; Troll, 1961; Woodward, 1987; Box, 2016). Because of this close relationship, climate‐based classification systems have been serving as surrogates for the continental and global biome schemes. Possibly the very first attempt of its kind can be ascribed to Merriam (1892, 1894). The work of climatologists such as Köppen (1900), Thornthwaite (1933, 1948), Köppen & Geiger (1954) and Bagnouls & Gaussen (1957), among many others, resulted in several bioclimatic classification systems that have had a major influence on understanding the global vegetation zonality (see Tukhanen, 1980 and Box, 2016 for selected reviews). Köppen (1900), placed paramount importance on plant distribution in identifying climatic regions, and his system is thus essentially a phytogeographical one. Two bioclimatic systems are directly relevant to the biome distribution, and both became true evergreens, appearing in every serious ecological textbook. These were Holdridge's (1947, 1967) and Whittaker's (1970) bioclimatic schemes (Fig. 1a,b). Holdridge's life zones relate the response of the vegetation to several interdependent environmental variables, namely temperature, precipitation and potential evapotranspiration. Whittaker based his two‐dimensional scheme on two simple climatic parameters – mean annual precipitation and mean annual temperature. Both systems fail to account, however, for climatic seasonality. Bailey's (1976, 1989) hierarchical system of ecoregions gained considerable traction in North America. An ecoregion is a large‐scale ecosystem (microecosystem; Bailey, 2005) delimited by macroclimate and various aspects of vegetation cover as well as several other (e.g. topographic, geologic, hydrologic) criteria pertinent to fine spatial scales.

### Physiognomy and the concept of vegetation formation

As one moves up the hierarchy of vegetation types, the floristic similarity among the types becomes less informative because shared physiognomic features gain in importance. Units compared over large distances share few species, yet the general appearance of vegetation (physiognomy) might remain very similar. This is based on the account of shared dominant growth forms, similar vertical layering, similarities in phenology, and indeed shared features of the macroclimate underpinning the compared vegetation formations.

The recognition of physiognomy as a comparative tool across large realms is most probably rooted in the so‐called ‘Buffon's law’ (de Buffon, 1761). This law suggests that, while vegetation classifications based on the dominant taxa might make sense regionally, they fail to do so when similar vegetation units from different continents are compared, because only a few taxa are shared (Shugart & Woodward, 2011).

Physiognomy has played a central role in the definition of Grisebach's (1838) concept of formation. Although hardly elaborated in the original publication, the formation was adopted by Schimper in his account of the vegetation of the world (1898; translated in 1903, and reproduced in the third edition in Schimper & von Faber, 1935). Schimper distinguished ‘edaphische Formationen’ and ‘klimatische Formationen’, recognizing the importance of climate and soils, respectively, as the major drivers of global vegetation patterns. As the physiognomy is the major determinant of the (plant) formation, so the formation is central to the concept of biome.

As noted by Mueller‐Dombois & Ellenberg (1974: 157), European ecologists followed the physiognomic focus (formation being characterized by dominant life form(s) that determine the overall appearance of vegetation), whereas American scholars defined the formation more geographically and climatically. These two seemingly different approaches are, in fact, complementary: macroclimate selects for the best‐adapted life forms that define a vegetation formation.

Several physiognomic systems have been suggested to classify plant cover (e.g. Dansereau, 1951; Küchler, 1956, 1966; Fosberg, 1961; Ellenberg & Mueller‐Dombois, 1967; Beard, 1973; Edwards, 1983). Some are crucial to understanding the biome concept, because the vegetation physiognomy is used as an indicator of biome identity. Unfortunately, the terms ‘vegetation physiognomy’ and ‘vegetation structure’ have often been used interchangeably (e.g. Küchler, 1966), introducing some confusion. Vegetation physiognomy (see earlier) refers to the overall appearance of vegetation cover (e.g. open woodland, forest, grassland). Physiognomy has its roots both in vegetation structure, which is spatially explicit as it is defined by vertical (layering) or horizontal (patchiness) aspects of vegetation patterning, and in vegetation texture (see Barkman, 1979 for a detailed discourse), defined by species composition or life‐form structure. Vegetation structure (as defined here) has not been used as a criterion in any known biome classification system. For instance, Küchler's (1956) system (see also Koeppe & De Long, 1958) is physiognomic, using a combination of a series of dichotomy criteria (woody vs herbaceous, broadleaf vs needleleaf, evergreen vs deciduous) and yielding 30 mappable physiognomic units. In America, Fosberg's (1961) popular system uses criteria such as openness of vegetation at the very top of the unit hierarchy and adopts the notion of formation at four ranks of the classification hierarchy (see also Ellenberg & Mueller‐Dombois, 1967; UNESCO, 1973; Mueller‐Dombois & Ellenberg, 1974).

Woodward *et al*. (2004) suggested that the biome concept can be supported by obvious physiognomic and phenological differences, for instance by considering evergreen vs deciduous behaviour, or broadleaf vs needleleaf foliage. Accordingly, five major tree‐dominated biomes can be recognized by satellites, based on leaf longevity and morphology, such as needleleaf evergreen, broadleaf evergreen, needleleaf deciduous, broadleaf cold deciduous and broadleaf drought deciduous biomes.

### Climate, soil, physiognomy and zonality: the first synthesis

Although the recognition of climate as a major driver of global biotic patterns and the phenomenon of global climatic zonality have almost prehistoric roots, the idea of soils showing a zonality patterning is much younger, yet nonetheless very influential. Schimper (1898) coined the well‐known climatic template of global ecology involving water availability and temperature, a template that I call ‘Schimperian world’. He has, however, also recognized edaphic factors of great importance, as captured in his concept of ‘edaphic formation’. Budyko (1974) coined the term ‘law of geographic zonality’ and ascribed the underlying concept to Dokuchaev (1899, 1948); see also Gerasimov, 1946). The essence of this idea is that zonal climate produces zonal soils, and these together define zonoecotopes (or eu‐ecotopes; ‘plakor’, *sensu* Vysotsky, 1909), which support zonal vegetation (‘periodic table of geographic zonality’: see table 31 in Budyko (1974) or an instructive summary in table 6 of Breckle (2002)).

Schimper and Dokuchaev set the scene for the zonality/azonality (Z/A) conceptual framework which was further elaborated (and applied worldwide) in the work of Heinrich Walter. The core element of the Walter's Z/A framework is the use of global bioclimatic zonation and global soil zonation as the basis for definition of his nine zonobiomes, ZB I–ZB IX (e.g. Walter & Box, 1976). Precipitation, temperature and seasonality patterns of both are the climatic criteria defining these zonobiomes.

**Figure 2:**
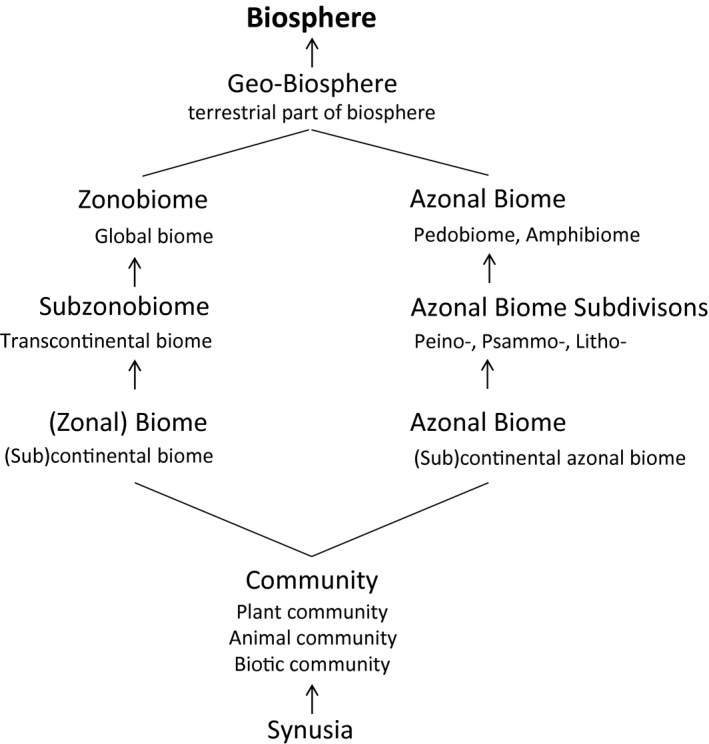
A modified classification scheme of zonal and azonal terrestrial biomes across subcontinental to global spatial scales; motivated by the original scheme of Walter & Box (1976). The bottom‐up succession follows the axis progressing ecosystem complexity and increasing spatial scale. Synusia is a functional (or spatial) subunit of a (biotic) community.

Naturally, the climate within every of those nine zonobiomes is not homogeneous. Rain shadows of mountain ranges, extreme latitudes and elevations, and offshore cold sea currents create new climatic niches by modifying the precipitation (and seasonality) and temperature conditions within a zonobiome. These niches support subzonobiomes, which, in most cases, also occur across several continents. Dry tropical forests (part of ZB I) and continental semideserts (part of ZB VII) may serve as examples such subzonobiomes. The subzonobiomes further subdivide into biomes in the strict sense (‘geographic units’ in Walter's terminology); these can then be called continental (or regional) biomes (Fig. 2). In effect, the biome classification hierarchy has three tiers.

The borders between zonobiomes are rarely sharp as the macroclimate changes continuously, often over tens or even hundreds of kilometres. Transitional areas (ecotones; see Shelford, 1945) – zonoecotones (ZEs) in Walter's terminology – are being formed, supporting vegetation of intermediate physiognomy; for instance, where boreal forest thins out to make space for tree‐less tundra (ZE VIII/IX), both deciduous and evergreen trees meet in the same community, such as in ZE IV/VI, or grasses codominate with low dwarf shrubs, as in ZE II/III.

Walter's Z/A system is not purely bioclimatic, however, as it also takes into consideration other ecological factors, most importantly soil (pedobiomes), water (pelobiomes, helobiomes, and amphibiomes), and elevation (orobiomes). These biomes are azonal. Leaning on Schimper's (1898) notion of ‘edaphic formation’, Walter (1942, 1970) and Breckle (2002) recognized a series of pedobiomes – ecosystems where soils would override the importance of climate and thus support peinobiomes on extremely nutrient‐poor soils (see, for instance, Mucina, 2018), psammobiomes on deep sand soils, or lithobiomes on rocky outcrops and pavements. Abundant water in (semi)terrestrial habitats, causing waterlogging or periodic flooding, is yet another factor driving functional and compositional change at the biome level and producing pelobiomes (mires and bogs) or helobiomes (temporarily flooded habitats). Mountain ranges spanning large changes in elevations, embedded within particular zonobiomes, create conditions for another deviation from the zonobiome standard. For those, Walter used the term orobiomes.

Walter's work formulates the contours of the first synthesis of the biome ecology, characterized by the operational definition of zonality in the context of global bioclimatic and soil classifications. Walter stood on the shoulders of several giants as he formulated his zonobiome concept, sourcing inspiration from:

1. the Schimperian view of the world where climatic factors (macroclimate) play the leading role and underpin ‘zonal formations’, while characteristics of soils underpin ‘azonal formations’ – emphasizing the role of plant physiology in shaping vegetation patterns;
2. the Russian geographical tradition and Dokuchaev's conceptual framework of geographic zonality of soils;
3. progress in climatology and the advent of comprehensive climatic classifications at the global level (e.g. Köppen, 1900; Thornthwaite, 1933).

### Multiple stable states of biomes

Vegetation is not a passive entity under the control of the environment. Across spatial scales, feedbacks between vegetation and climate, soils and disturbance regimes create new environments. These feedbacks, assisted by disturbance factors such as fire and mega‐herbivore grazing, create the potential for the emergence of multiple biome states that are stable over long time series (multiple stable states (MSS; Charles‐Dominique *et al*., 2015; Moncrieff *et al*., 2016) or alternate/alternative stable states (e.g. Warman & Moles, 2009; Cramer *et al*., 2018)). The existence of MSS implies that a system, when disturbed from one state to another, does not return to its original state once the cause is removed; however, a second factor takes over and holds the system in the new state for a long time (Dublin *et al*., 1990).

Along with fire (e.g. Dantas *et al*., 2016), herbivores also facilitate stabilizing feedbacks and transitions between MSS (e.g. Dublin *et al*., 1990; Moncrieff *et al*., 2016). The impact of grazing on the changing physiognomy of biomes has been documented in many studies (e.g. Dublin *et al*., 1990; Hempson *et al*., 2015; Charles‐Dominique *et al*., 2016), as has the effect of fire (e.g. van Wilgen *et al*., 2003; Bond *et al*., 2005; Lehmann *et al*., 2014). Megaherbivore grazing and fire are perhaps the most obvious large‐scale disturbance factors shaping the appearance and dynamics of vegetation cover. They can be considered as two sides of the same coin (Bond & Keeley, 2005) in the context of large‐scale and persisting disturbance, as their interactions have a major impact on community assembly and physiognomy (tree establishment and abundance, grass cover dynamics) through control of population and community dynamics and processes (e.g. Dublin *et al*., 1990; Van Langevelde *et al*., 2003; Staver *et al*., 2009; Radloff *et al*., 2014). From a deep‐time perspective, on the other hand, exclusion of megaherbivores (often associated with a change in fire regime) can lead to both physiognomic changes within a biome or, in extreme cases, demise of the biome. The impact of notorious faunal megaextinctions in formerly isolated or human‐free ecosystems (e.g. Burney & Flannery, 2005; Gill *et al*., 2009; Young *et al*., 2016) is the epitome of profound changes to biome physiognomy on a large scale as a consequence of missing major ecological engineers. Bush encroachment, possibly documented for the first time by Walter (1954a) as involving a relatively rapid change from grass‐dominated to woody‐dominated systems, is yet another phenomenon of this kind. The underpinning reasons for this profound physiognomy change linking various states of biome appearance are still the subject of debate (e.g. Bond & Midgley, 2000; Roques *et al*., 2001; Stevens *et al*., 2017).

**Figure 3:**
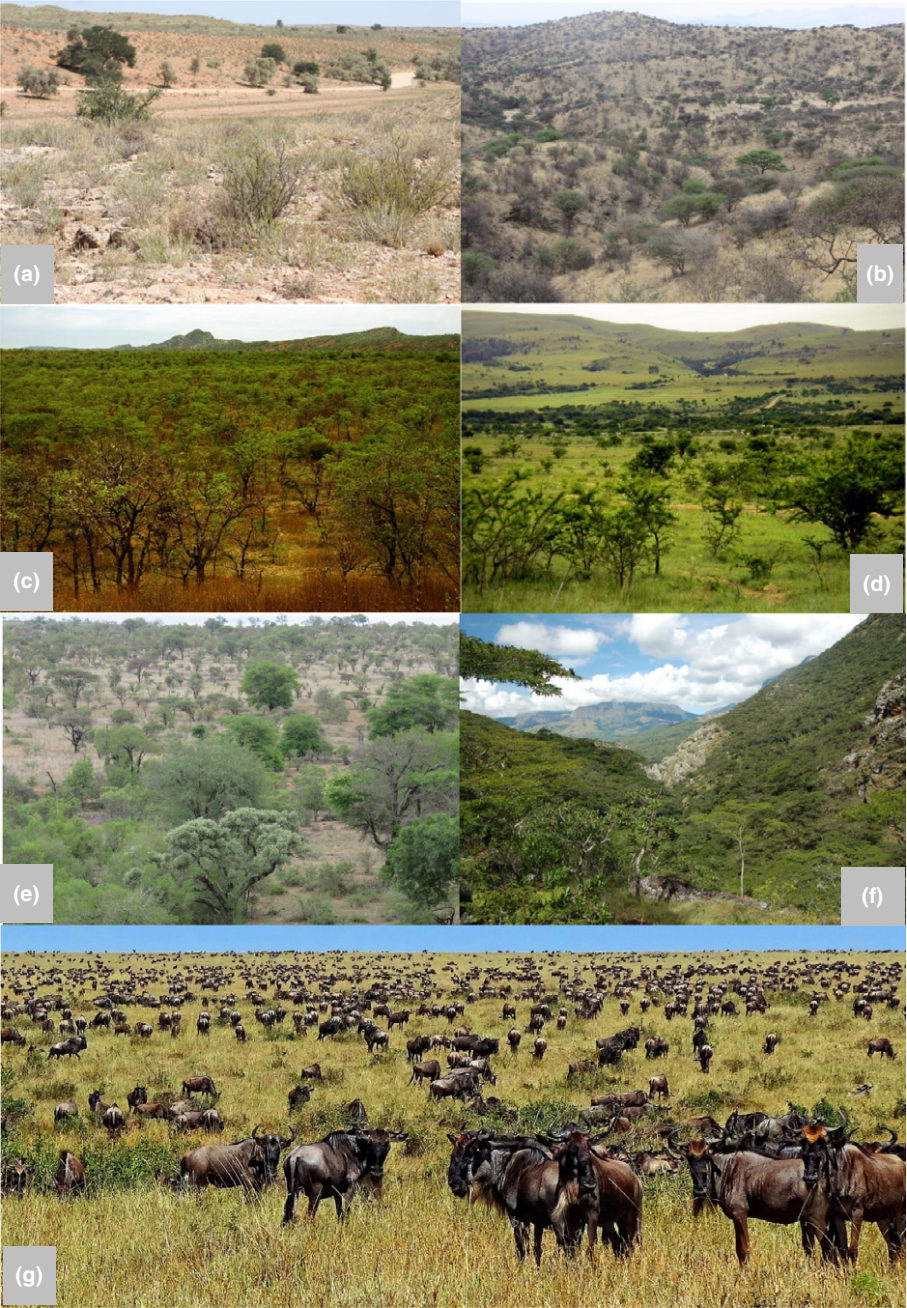
Physiognomic aspects of the African savanna. (a) Arid savanna grasslands with scattered scrub of the southern Kalahari, Khalaghadi Transfrontier Park, South Africa: partly stabilized dunes and calcrete outcrops fringing dry bed of the Nossob River. (b) Central Bushveld of Central Namibia: open savanna woodland dominated by *Senegalia* (*Acacia) hereroensis* in the Danie Viljoen National Park near Windhoek. (c) Woodland dominated by mopane (*Colophospermum mopane*): South Africa, Honnet Nature Reserve neat Tsipise, Venda. (d) Subescarpment *Vachellia* (*Acacia) karroo* thornveld with intensively grazed C4 grassland understorey, near Butterworth, Eastern Cape, South Africa. (e) Lowveld savanna woodlands of the Kruger National Park, South Africa, dominated by broadleaved Combretaceae. (f) Miombo woodlands (dense seasonal forest) with *Brachystegia boehmii, Brachystegia microphylla*,* Brachystegia spiciformis*,* Uapaca kirkiana* and *Vangueriopsis lanciflora,* Zimbabwe, Chimanimani Mountains. (g) Iconic Serengeti savanna grasslands; blue wildebeest (*Connochaetes taurinus*) migrating to Masai Mara Game Reserve (Kenya). Photo credits: (a–e) L. Mucina; (f) M.C. Lötter; (g) B.C. Tørrissen ( http://bjornfree.com/galleries.html).

Walter has not, implicitly, considered large‐scale, natural disturbance as part of his conceptual biome scheme. This has been remedied by Rutherford *et al*. (2006), who expanded the classical Schimperian world in their biome account of South Africa, by adding disturbance as an important dimension of the biome concept. Rutherford *et al*. (2006) discussed the physiognomy of several biomes and established that a biome can occur in the same region in several forms, showing large variability in dominance of life forms and vertical structuring. Not surprisingly, in the South African context, these biomes are fynbos (a Mediterranean‐type scrub) and savanna – both structured by natural recurrent fires, in combination with megaherbivore pressure. Bond (2005) called such biomes ‘consumer‐controlled’ African savanna, covering more than half of the area of the continent, and they can appear as pure grassland, dry grassland with scattered shrubs, open tree/grass‐dominated woodland and semi‐closed forest with grass layer understorey (Fig. 3). Besides regional climatic deviations and soil characteristics, much of this variability is based on a plethora of disturbance regimes stabilizing MSS (e.g. Osborne *et al*., 2018).

## Going functional

A large geographic area (LGA), defined as (a substantial portion of) a continent and characterized by well‐defined macroclimatic patterns, is expected to be heterogeneous in terms of geology and soils. These, together with the macroclimate and regional/local mesoclimatic and microclimatic conditions, create an intricate network of environmental gradients along which regional species pools are being selected across meta‐community networks (defined as complexes of communities interconnected by flow of energy and matter; see also Leibold *et al*., 2004). Large‐scale disturbance imposes yet another important environmental filter.

Each of these species contributes to the total set of functional traits (trait pool) that is subject to environmental sorting along ecological gradients. The sorting has an impact on biotic interactions that, in a reverse manner, modify the sorting processes. The trait pools of the local communities are combined to form metacommunity trait pools (defined as the sum of all traits of all species across all local communities of an LGA). Processes of environmental filtering and biotic interactions modify these trait pools by selecting for species assemblages along environmental gradients within the given metacommunity framework.

The traits and trait pools determine community patterns and functioning. Knowing which plant traits are dominating the ecological functions in a landscape dominated by terrestrial vegetation is the major prerequisite to successful predictive modelling of the functional biomes.

Traits *per se* are highly informative (predictive) in many ecological responses (see Lavorel & Garnier, 2002 for a conceptual framework), yet ‘the usefulness of traits in predictive models hinges on deepening our understanding of which traits drive ecological processes at organismal, community, and ecosystem scales’ (Funk *et al*., 2017). Global scales call for simplification, for example by focusing on functional syndromes rather than species‐based approaches. Indeed, functional syndromes (Lavorel *et al*., 1997) are proving to be effective tools for solving problems of biome ecology, in both predictive and retrodictive modelling of mechanistic as well as functional biomes.

### Biome modelling: the second synthesis

The building of predictive (and retrodictive) biome models was a logical outcome of convergence of several pieces of scientific theory, including: recognition of scale and scaling up of ecological processes from individual to large‐scale ecosystems; recognition of the hierarchical nature of the environmental determinant of biotic community patterns; the formulation of zonal/azonal theory; achievements of global climatology and soil science (underpinnings of climate–vegetation interactions, link between zonal climate and zonal soils, understanding of global primary productivity dynamics, and accumulation of large spatial datasets); deeper understanding of global cycles of matter and energy, assisted by technological breakthroughs in the field of spatial data handling (GIS and related software development in the first place); hardware development, including computational power; and automation of data collection using remote‐sensing technology. In summary, the biome modelling was driven by both scientific theory and technological progress.

Use of bioclimate as a proxy for delimitation of a biome distribution can be seen as a primordial form of biome modelling. If life forms determine physiognomy and these life forms carry ecological signals, then defining the link between the distribution of the life forms and bioclimate would be the first logical step in modelling their spatial distribution. Shugart & Woodward (2011) observed that probably the most highly elaborate model coupling climate conditions with plant attributes and aimed at predicting patterns of global and regional vegetation was developed by Box (1981a,b). The Box scheme combines the putative convergence of physiognomy observed in taxonomically unrelated plants in equivalent environments with the concept of vegetation type determined by combinations of dominant taxa. The crucial assumption of his modelling approach is the link between the plant functional types (PFTs) and climate, using physiological limits to distribution of the PFTs as constraints.

**Figure 4:**
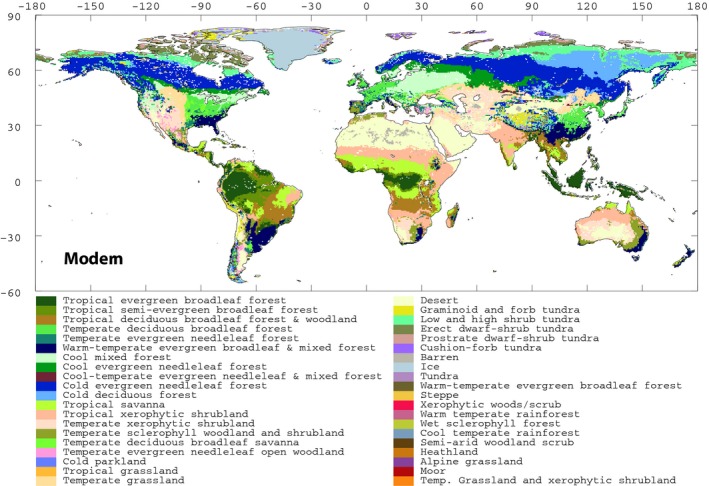
Biomes of the world as modelled by BIOME4 (Kaplan *et al*., 2003) – a typical example of an equilibrium vegetation model. Reproduced from: Paleoclimate Modeling Intercomparison Project II ( http://pmip2.lsce.ipsl.fr/synth/biome4.shtml).

Box's work punctuates the onset of the second synthesis of the biome ecology, as it sets the scene for development of equilibrium modelling frameworks (e.g. Prentice *et al*., 1992), further improved by increasingly complex and sophisticated models, including coupled biome distribution/biogeochemistry models (e.g. Woodward *et al*., 1995; Kaplan *et al*., 2003), dynamic global vegetation models (e.g. Foley *et al*., 1996; Cramer *et al*., 2001), and earth system models (e.g. Hill *et al*., 2004). It is beyond the scope of this review to analyse the pros and cons of all these modelling frameworks (for detailed reviews, see Zhou & Wang, 2000; Cramer, 2002; Tang & Bartlein, 2008). Some of these models, such as BIOME4 of Kaplan *et al*. (2003; Fig. 4), are remarkably predictive in mimicking the distribution of biomes using other approaches.

In addition to the obvious merits of the application of predictive biome modelling in climate change research (e.g. Woodward *et al*., 1995; Cramer, 2002; Gonzalez *et al*., 2010; Donoghue & Edwards, 2014), it appears equally fruitful to use this methodology in retrodictive modelling aimed at the reconstruction of biomes. Reliable climatic proxies proved useful in predicting biomes in the Holocene and Late Pleistocene (e.g. Foley, 1994; Midgley *et al*., 2001). The reconstructed palaeoclimate data, when combined with fossil archives (e.g. Prentice & Webb, 1998; Ni *et al*., 2010), for instance, proved effective in deep‐time reconstructions (e.g. the Neogene, Jacques *et al*., 2013; the Miocene‐Pliocene, Salzmann *et al*., 2008).

### Process‐based functional biomes

Patterns are an outcome of processes. The diversity of biomes should be seen as a result of multiple processes operating at multiple spatial and temporal scales. Fine‐scale processes (such as plant growth) rule the large‐scale dynamics of ecosystem productivity, which, in turn, shapes large‐scale patterns. Hence, knowing the nature of fine‐scale processes and the nature of the players involved, namely the species pools defining flora which, in turn, define trait pools and trait spaces, it should be possible to employ the hierarchically structured processes to predict processes in hierarchically structured patterns.

The success of using fine‐scale processes (e.g. photosynthetic pathways, plant growth parameters) to predict biome‐scale patterns (physiognomy and trait pools) very much depends on the ability to up‐scale the fine‐scale processes and to devise indices that best inform the dominating functions shaping the dynamics and physiognomy at the biome level. Ecosystem productivity, expressed as net primary productivity (NPP; Lieth, 1964) or normalized differential vegetation index (NDVI), carries a promise of such ‘indices’, especially after Michaletz *et al*. (2017) successfully demonstrated that variation in NPP across global climate gradients primarily reflects the influence of climate on growing season length and stand biomass, as well as stand age. NDVI can be captured, for instance, by remote sensing and can therefore assist in large‐scale mapping of functional large‐scale community patterns, such as biomes. NDVI summarizes primary productivity, which is, in turn, a function of extrinsic factors, such as resource availability (supplied by both climate and soils), and intrinsic factors, such as evolutionary constraints on growth.

The ecosystem functional type (EFT) concept (e.g. Paruelo *et al*., 2001; Alcaraz‐Segura *et al*., 2006) is perhaps the first serious attempt to group ecosystems (at large scales) on the basis of shared functional behaviour, defined by sharing similar dynamics of matter and energy exchanges between the biota and the physical environment. The EFT approach uses time series of spectral vegetation indices to capture the carbon gain dynamics, considered to be the most integrative indicator of ecosystem functioning. Satellite‐derived dynamics of primary production is used as the source of calculations of the indices. NDVI was also used by Buitenwerf *et al*. (2015), who examined NDVI time series and classified land surface into ‘phenomes. Higgins *et al*. (2016) criticized the biome schemes based on climate and proposed a new biome map that would use information on vegetation productivity index (based on NDVI), the link between vegetation activity and the driest or coldest part of the year, and vegetation height. These three indices were supposed to underpin an alternative classification scheme for comparing biogeochemical rates of terrestrial ecosystems.

The attempts so far (Paruelo *et al*., 2001; Buitenwerf *et al*., 2015; Higgins *et al*., 2016), when corroborated by equilibrium‐coupled predictive biome modelling (Kaplan *et al*., 2003), have shown promising results in predicting zonobiome patterns. There is, however, still a lot of latitude for improvement in predicting fine‐scale patterns of azonal biomes as well as consumer‐driven biomes (see section on ‘Anthromes: the human‐made biomes’). I suggest that the current limitations of the functional‐biome modelling are less of a theoretical nature (selection of the correct indices reflecting the functions underpinning azonal patterns), and more to do with lack of availability of fine‐scale spatial datasets (on climate, soil patterns, hydrology and large‐scale disturbance) of high resolution.

The use of PFTs, considered ‘crude’ groupings, invited much criticism from those who argue that not only trait syndromes, but also traits (and their intratrait variability) should be considered, as demonstrated or argued by Pavlick *et al*. (2013), Scheiter *et al*. (2013), Sakschewski *et al*. (2015) and Moncrieff *et al*. (2016). The latter authors see progress in the modelling of global vegetation in the acknowledgement that vegetation patterns are not deterministic in relation to climate, consider processes producing the feedbacks responsible for multiple stable states, and explicitly explore the role of vegetation history in model building.

If we wish to define biomes in functional terms (and terms that reflect the evolutionary assembly as well, including local species radiations, extinction patterns, migrations), then predictability of such biome structures would very much depend on choosing the most informative functional traits or trait syndromes – the key to understanding the biome as a functional entity. Because of the large‐scale nature of biomes, information on patterns and their variability of such traits at the biome spatial scale is of vital importance. In this context, Reich & Oleksyn (2004; see also Reich, 2005) studied global patterns of leaf nitrogen (N) and phosphorus (P) in relation to temperature and latitude. Dahlin *et al*. (2013) and van Bodegom *et al*. (2014) produced global maps by projecting trait–environment relationships for three plant functional traits (leaf mass per area, stem‐specific density and seed mass). Butler *et al*. (2017) were able to map how several plant traits, closely coupled to photosynthesis (specific leaf area, dry mass‐based concentrations of leaf N and P), vary within and among biomes over a grid of cells covering the entire vegetated land surface**.** Yet, upscaling of the assembly functions of these traits to large spatial scales (biome level) still seems to be out of reach, despite their useful role as ‘supertraits’ (for a definition, see Madin *et al*., 2016) able to capture a large amount of process variation. I suggest that our understanding of the causal links between trait patterns and vegetation patterns is in its infancy. However, Enquist *et al*.'s (2015) trait driver theory, attempting a synthesis of trait‐based and metabolic scaling approaches, is a step in right direction, as it shows that the shape and dynamics of trait and size distributions can be linked to fundamental drivers of biotic communities.

## Going evolutionary

The biome (as any biotic community) is a result of assembly processes at many scales of spatial and temporal complexity. Focusing on long‐term evolutionary processes shaping both the taxonomic and trait pools, we can view biomes as ‘theatres of evolution’ (Moncrieff *et al*., 2016), where species are born and die, and into and out of which they immigrate and emigrate, in response to both fast and slow changes in environmental conditions, leaving environmental filters to select the fittest biota. Progress in trait‐focused functional plant ecology and palaeogeography, and new developments in genomics open new ways of inferring the patterns of the past, enhancing our understanding of the underpinning mechanisms of biome changes.

There are several hotspots in the current evolutionary biome ecology (several of which I mention), offering a cross‐section of the current activities in the field. In the following, I shall address (see the section ‘Ante prima’) the interface between biome ecology and biogeography (see the section ‘Drivers of community patterns at large spatial scales’), the phenomenon of legacy biomes and (see the section ‘Going functional’) trait and trait–syndrome convergence/divergence phenomena in the framework of evolutionary biome assembly across and within continents. Biome ecology is profiting from a new, emerging interface between ecology and evolution – an interface offering space for the third synthesis (see the section ‘Going evolutionary’ later) and allowing biome ecology to leap from a pattern‐focused to process‐focused scientific discipline.

### Biome ecology and historical biogeography meeting

Shugart & Woodward's (2011) subchapter entitled ‘Early environmental biogeography: from mapping plant species distribution to mapping vegetation’ sets the scene of this section of my review: the nature of the relationship between classical biogeography focused on distribution of species (or groups thereof) in large natural (e.g. islands) or artificial (spatial grids) units, and one of the major focus areas of ecology: understanding of the spatial patterns of plant communities reflecting environmentally and spatially well‐defined, and hence homogeneous, habitats and habitat complexes.

The distribution areas of plants are unique, but some species show a high level of spatial similarity which can be exploited for the definition of ‘biogeographical coincidence’ underpinning concepts such as the geoelement (Meusel *et al*., 1965) and phytochorion (Takhtajan, 1986), including floristic kingdoms, regions and the like. These biogeographical realms/regions (also called phytochoria or zoochoria) are based on distributional data and/or shared evolutionary history (e.g. in terms of phylogenetic origin; Holt *et al*., 2013; Daru *et al*., 2017, 2018). Plant species also co‐occur in habitats where they assemble into plant communities; these also occupy space (have distribution area). Species’ co‐occurrence patterns can be mapped (e.g. von Martius, 1831; De Candolle, 1855; Linder *et al*., 2012) and so can plant communities (Grisebach, 1872; Lavrenko & Sochava, 1954; Bohn *et al*., 2003; Mucina & Rutherford, 2006; Lillesø *et al*., 2011, and many more) and this makes vegetation a subject of biogeographical enquiry.

Understanding spatial and temporal distribution of the functions of living organisms and of the resulting ecosystems is adding a new dimension of biogeographic enquiry and sets foundations of functional biogeography (Reichstein *et al*., 2014). Functional biogeography is thus becoming the meeting ground of biogeography and biome ecology. The phytochoria of biogeography and mapping units of vegetation ecology are two different concepts, yet this difference is becoming less obvious while progressing towards large spatial scales. Biogeography and vegetation ecology were, seemingly, going their own ways – producing biogeographic spatial divisions and vegetation maps, respectively. The relationship between phytochoria and large‐scale vegetation mapping units is still a rather dormant field of the biogeography–ecology interface. Yet, some earlier cross‐pollination between vegetation mapping and biogeographic land classifications did result in informative large‐scale schemes. The influence of John Beard's vegetation mapping of Western Australia (Beard, 1975) on the Interim Biogeographical Regionalisation for Australia 7 (Department of the Environment and Energy, 2017) can serve as an example. More recently, the study of Silva de Miranda *et al*. (2018) used tree distribution areas to delimit biomes in lowland tropical South America.

### Legacy biomes

It is common to find ‘odd’ habitats supporting very different biota when compared with the biome dominating the landscape matrix. Fire‐prone savannas or grasslands may contain pockets of forests, and green oases are found in many deserts. If not driven by edaphic or hydrologic conditions, these may be considered as witnesses of past climatic conditions – they are relict biotic communities dwelling in refugia (Keppel *et al*., 2012) that might not have reached equilibrium in the currently prevailing environmental conditions (e.g. Svenning & Skov, 2007; Moncrieff *et al*., 2014b, 2016). Ackerly's (2003; see also Crisp, 2006) term, ‘no analogue’ communities, would fit the bill well.

In southern Africa, the warm‐temperate forests form an archipelago of fire‐shy patches embedded within fynbos and grassland biomes (Mucina *et al*., 2006), representing a relic of a Miocene‐Pliocene forest/woodland biome. In the Top End of Australia, home to extensive eucalyptus‐dominated tropical woodlands, patches of tropical dry seasonal forests (Webb *et al*., 1986; Bowman, 2000; Ondei *et al*., 2017) – mesic island in the sea of fire – are found. Some of the palaeorefugia experience dramatic dynamics of expansion and retraction, reflecting the palaeoclimatic patterns of the Plio‐Pleistocene as established, for instance, in the tropical rainforest of northern Queensland, Australia (e.g. Moritz, 2005; VanDerWal *et al*., 2009). Europe is home to many examples of neorefugia, such as those found on summits of European nemoral mountain ranges, which are home to extrazonal fragments of Pleistocene cold‐climate biomes, represented by mountain tundra and nival deserts.

All these instances of legacy biomes are posing a serious challenge to the predictive (or retrodictive) modelling at the biome level, not only because of their small size and patchy occurrence, but also, in particular, due to environmental conditions grossly deviating from the macroclimate that are often difficult to capture in fine‐scale manner.

### Convergence and divergence: failed agenda?

Physiognomic similarity between various ecosystems residing on distant continents is an old and well‐established biogeographic story (e.g. Grisebach, 1872; Schimper, 1903; Cain, 1950). This similarity, based on the report of similar mixes of the same suite of growth forms found under similar macroclimatic conditions, is contrasted with an almost complete lack of shared species and even genera. The putative physiognomic and functional convergence among the five known Mediterranean‐type regional biomes (e.g. Naveh, 1967; Specht, 1969; Mooney & Dunn, 1970; Cowling & Campbell, 1980) located in ethesial climatic regions comes to mind. Large‐scale scientific cooperative projects such as the International Biological Programme (IBP 1964–74) used the convergent phenomena as the basis of cross‐continental comparisons and searches for commonalities (e.g. Cody & Mooney, 1978; Shmida & Whittaker, 1984; Shugart & Woodward, 2011).

Moncrieff *et al*. (2016) observed that intercontinental comparisons between environmentally similar, yet floristically disparate regions (e.g. focusing on continental/regional biomes within a well‐defined zonobiome; Dantas & Pausas, 2013; Moncrieff *et al*., 2014a; Forrestel *et al*., 2017) revealed that the assumed pervasive environmental filtering, supposed to result in deterministic patterns of vegetation structure and function in relation to prevailing environmental conditions (hence convergence), is seldom realized. This is undoubtedly pointing to a strong biogeographical contingency. Indeed, regional (continental) idiosyncrasies in trait compositions across forests and savanna biomes have been well documented (Lehmann *et al*., 2011, 2014). This observation suggests that the structural responses of savannas to variation in climate, soil and disturbance also vary among continents (Moncrieff *et al*., 2015), leading to differences in the environmental limits of savannas across continents (Moncrieff *et al*., 2016). Interestingly, Lusk *et al*. (2016), when comparing temperate rainforests of New Zealand and South America, found that environmental filtering has produced similar values of individual traits, yet only partial convergence of functional trait combinations was detected.

The quest to understand the putatively similar physiognomy between regional biomes (across continents) in the 1960s and 1970s of the last century failed to reveal the underlying mechanisms, although traits and syndromes have been used as tools. I suggest, however, that the last word about the convergence has not yet been heard. There are two issues that need to be addressed, namely the proper choice of comparable objects to be subject to convergence/divergence tests, and the proper tools used to test this phenomenon. In this context, I suggest that a careful distinction is made between zonobiomes (global), subzonobiomes (transcontinental) and biomes (regional–continental; see the previous paragraph for support). Also, there is a set of tools designed to test for convergence/divergence that were developed previously (Orlóci, 1971; Orlóci *et al*., 1986; Pillar & Orlóci, 2004) but have not yet been used in the biome context. This approach implies hierarchical nestedness of the character (trait) sets and uses information‐statistical tools to infer convergence/divergence across the hierarchy. The tools, originally designed to test trait patterns along local ecological gradients (Ackerly & Cornwell, 2007; Pillar *et al*., 2009; Pillar & Duarte, 2010), when linked to large‐scale trait databases, may also prove useful at biome scales.

### Evolutionary biome assembly: the third synthesis

Biomes undergo assembly and disassembly like other communities at finer spatial temporal scales. I suggest that slow‐acting evolutionary processes, such as speciation/extinction and migrations, set the stage for the classical community assembly drivers operating on short timescales. The evolutionary processes, constrained by macroclimatic as well as geological and hydrological landscape fabrics, create biogeographic species pools (Carstensen *et al*., 2013). These are the substrate for abiotic filtering and limiting similarity to select the regional (ecological) species pools (Zobel, 1997), which serve as the source of local species pools that characterize local biotic communities at the habitat level. If we are to disentangle the speciation/extinction and migration history of major microbial, plant and animal clades, we will need deep insights into the assembly of biota in large geographical regions, indeed into the evolution of biomes.

Shugart & Woodward (2011) asked the question, ‘How much does the evolutionary history of a region affect the life forms found there?’ Higgins *et al*. (2016) suggested that the premise behind biomes is that the environment selects over evolutionary time and filters over ecological time for vegetation attributes, producing a globally coherent distribution of plant structure and function – hence biomes. Biome niche conservatism (Crisp *et al*., 2009; Donoghue & Edwards, 2014) offers essential supportive evidence underpinning this coherence by recognizing that the biome boundaries can represent formidable constraints on the adaptive radiation of lineages (Moncrieff *et al*., 2016).

How do we approach the evolutionary biome assembly? Crisp (2006), using tools of historical biogeography and molecular phylogenetic information, offers a research agenda by listing several specific questions relevant to biome assembly, including: what is the geographical pattern of sister taxa among biomes; is there a prediction of a congruent pattern resulting from historical events (such as vicariance or opening of a migration corridor); if migration is inferred, what was its direction between biomes; and did the timing of divergence events coincide in taxa for which congruence is inferred?

**Figure 5:**
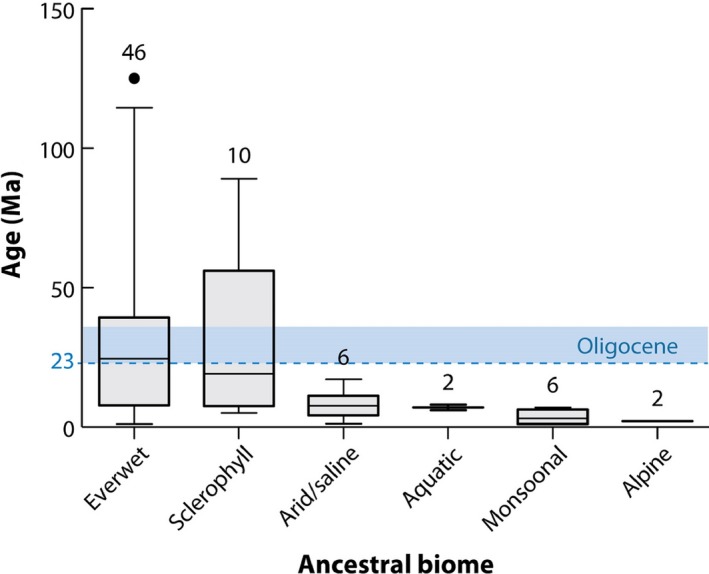
Ages of crown‐groups of selected lineages of Australian flora grouped by six inferred ancestral ‘biomes’, showing that the lineages that originated in the ‘sclerophyll’ (Mediterranean‐type woodlands and scrub biome) and ‘ever‐wet’ (rainforest) biomes are the oldest. The blue bar represents the Oligocene (34–23 million years ago (Ma)). The boxes show the median and the 25^th^ and 75^th^ percentiles, whereas the error bars show the 2.5^th^ and 97.5^th^ percentiles; *n* is the number of lineages analysed per ‘biome’. The alpine biome includes temperate grassland. (Source: Crisp & Cook, 2013: Fig. 8; reproduced with permission of the Annual Reviews Inc.). [Correction added after online publication 27 November 2018; the figure has been replaced with the correct figure; the figure legend is unchanged.]

In the past, the research agenda focused on the assembly of regional flora, and it was a domain of classical descriptive biogeography, assisted by classical palaeobiology. Modern genomics opens new avenues and creates new data archives that could potentially tackle all questions as listed by Crisp (2006). Interestingly, the most informative and convincing meta‐analyses involving multiple clades come from regions characterized by landscape‐evolutionary antiquity, such as old stable landscapes (Hopper, 2009; Mucina & Wardell‐Johnson, 2011). For instance, Linder (2003) found that about half of the species‐rich flora of the Capensis are a result of *c*. 33 species radiations, most of them being recent rapid radiations. Verboom *et al*. (2009), using a comparative framework of two neighbouring, ecologically contrasting biomes (Mediterranean‐type fynbos vs warm‐temperate semidesert Succulent Karroo) and looking into evolution of 17 plant groups shared by those biomes, were able to establish that Fynbos (home to older clades) had a major impact on the assembly of flora of the younger Succulent Karoo. A continental meta‐analysis of Australian clades (Crisp & Cook, 2013) identified the sclerophyllous woodlands/scrub and tropical rainforests as the evolutionary oldest biomes in Australia (Fig. 5). Insightful studies by Simon *et al*. (2009), Pennington *et al*. (2006, 2009), Hoorn *et al*. (2010), Hughes *et al*. (2013), Eiserhardt *et al*. (2017), Antonelli *et al*. (2018), Kaya *et al*. (2018) and Slik *et al*. (2018), to mention but a few, shed light on the evolution of tropical (both grass‐rich and tree‐dominated) biomes.

Technology‐driven progress in evolutionary biology and geography (GIS), big data (larger and better accessible archives of relevant genomic and geographic information) and progress in palaeogeography are creating a common, exciting interface where historical biogeography, palaeobiology, functional ecology and genomics meet. These developments herald the coming of the third synthesis of the theory of biomes, based on macroecology and macroevolution. There's still a long way to go, but a loud opening statement has already been made by Moncrieff *et al*. (2016).

## Anthromes: the human‐made biomes

No organism has had such a profound impact on the composition and dynamics of biotic communities as the human race. The biome composition on our planet is under steady change. Humans have only accentuated these natural changes and, in some cases, created conditions for the emergence of new environmental niches and through (un)intentional support fostered biological invasions. Humans protect, modify and destroy. Modifications resulting from human use of natural resources may lead to the formation of ‘novel ecosystems’ (Hobbs *et al*., 2006; Morse *et al*., 2014) or, in more Eurocentric terminology, synanthropic or anthropogenic (= human‐made) habitats supporting new combinations of species. These modifications create anthropogenic biomes (also called ‘anthromes’; Ellis & Ramankutty, 2008).

## How to be a biome?

**Figure 6:**
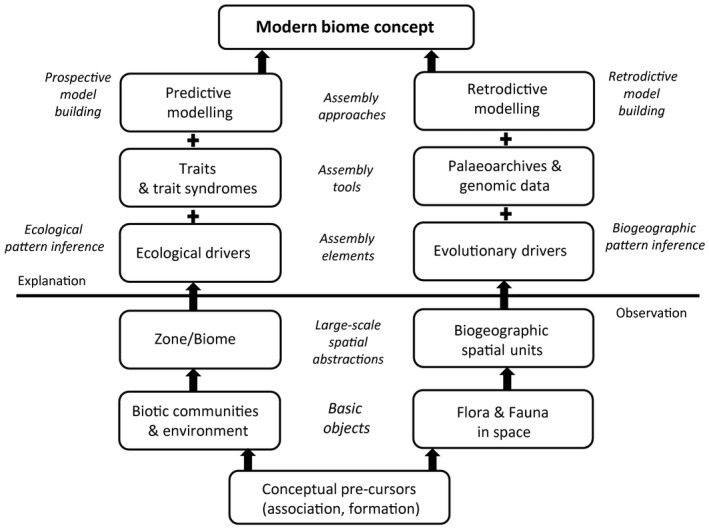
A scheme of the conceptual evolution of a biome, involving basic objects, abstractions, elements, tools, and approaches of ecological (left lane) and evolutionary (right lane) pathways, both progressing from the observational to the explanatory phase of progress towards the modern understanding of the biome.

The concept of a biome underwent an intricate scientific evolution, involving two major streams (Fig. 6): ecological (dominated by development in community ecology) and evolutionary (dominated by biogeographic ideas and tools).

There are several ways in which to approach biome construction (Table 2). Each has its merits, yet none of them offers a comprehensive recipe on how to do so. The physiognomy of plant formation would be straightforward, yet it does not necessarily reflect the functionality of the broad‐scale biotic community. The existence of multiple stable states known for some biomes, especially those that are consumer‐dominated, invalidates the physiognomy as the key to biome recognition. Climate is a much better predictor, but perhaps only at the zonobiome and subzonobiome levels. It undoubtedly fails as a sole predictor across more than one continent, as continental/regional biomes show large climatic variability. Climate also fails in predicting MSS and legacy biomes. At any scale, azonal biomes can hardly be predicted by climate altogether. Classical modelling linking PFTs, their ecophysiological limits and knowledge of biogeochemical cycles is a robust tool at the large scale of zonobiomes, but still has not delivered in the case of fine‐scale azonal biomes. Functional modelling (involving ecosystem functions, and perhaps also linked to functional trait composition) offers a very exciting perspective in defining the biomes as functional units. There is, however, some way to go to identify most informative mixtures of functional surrogates to serve the modelling effectively. Biogeographical (chorological) spatial units and biomes show a large degree of overlap, yet species co‐occurrence does not account for ecological functionality. Using vegetation classification and mapping schemes as a tool of biome delimitation offers new perspectives on more precise spatial and ecological delimitation of biomes. A successful translation of a vegetation typology into a biome typology requires knowledge of the major drivers underpinning the vegetation patterns at large spatial scales.

**Table 2:** Tools of delimitation (construction and prediction/retrodiction) of biomes. The term ‘physical environment’ includes climate, soil and hydrological characteristics

Approach	Criteria of delimitation	References
Physiognomy	Appearance of vegetation (combination of growth forms)	Grisebach (1838), Küchler (1949), Fosberg (1961), Ellenberg & Mueller‐Dombois (1967), UNESCO (1973)
Climate (only)	Using temperature and precipitation (expressed by various indices) as predictive of biome patterns	Merriam (1894), Köppen (1900), Thornthwaite (1933), Holdridge (1947), Whittaker (1970), Schultz (2005), Hobbs & Mcintyre (2005), Rivas‐Martínez *et al*. (2011)
Physical environment	Combined climate (zonal units) and soil/water (azonal units) as drivers of biome patterns	Schimper (1898), Walter (1954a,b)
Physical environment + disturbance	Recognition of multiple stable states as a result of vegetation–environment feedback and disturbance	Dublin *et al*. (1990), Bond (2005), Rutherford *et al*. (2006)
Vegetation patterns	Lumping of vegetation classification units (= redefining a vegetation map) to create a biome scheme	Rutherford *et al*. (2006), Mucina (2013), Mucina *et al*. (2016)
Species distribution coincidence	Biomes are the same as phytogeographic units	White (1983), Crisp (2006), EMPRABA (www.empraba.br)
Eclectic	Biome defined as lump sum of ecoregions that were defined on manifold (yet often unclear) criteria	Olson *et al*. (2001)
Functional ecosystem characteristic	Net primary productivity, normalized differential vegetation index, and production‐focused ecosystem characteristics related to model biomes	Lieth (1964), Paruelo *et al*. (2001), Scheiter *et al*. (2013), Higgins *et al*. (2016)
Physical environment + plant functional types + physiology (+ biochemical cycles)	Classical equilibrium‐coupled and dynamic global vegetation biome modelling (including modelling involving biochemical fluxes)	Box (1981a,b), Prentice *et al*. (1992), Kaplan *et al*. (2003), Sitch *et al*. (2003)
Physical environment + functional traits + evolutionary assembly	Linking current and past patterns in a comprehensive macroevolutionary and macroecological framework	Moncrieff *et al*. (2016)
Human impact	Special category of biomes (anthromes) defined as human constructions (arable fields, timber plantations, human settlements and land communication structures)	Ellis & Ramankutty (2008)

The ecological line of development involves recognition of global patterns of similarity between vegetation types, formulation of a framework of ecological drivers and upscaling of the physiology of plants as a basis for predictive modelling. The pervasive change of ‘ecological language’ from species to functional traits and syndromes is underpinned by the assumption that traits carry better ecological signal than the taxonomic identity. Knowledge of ecological drivers and trait (syndromes) patterns allows the prediction (and reconstruction) of biome patterns.

Along the evolutionary line of development, the biome concept could profit more from classical biogeography by using the distribution areas of constituent plants (and animals) to define units of spatial congruence. Palaeoarchives and modern genomic tools that assist inquiry into cladogenesis, as well as into the evolution of traits and ancestral distribution areas, are powerful tools enhancing our understanding of the dynamics of biota at global, continental and regional biome levels. Linking the fine‐scale genomic information with large‐scale phenomena such as biomes is poised to offer new perspectives on how the biomes have assembled.

The biome is and remains a key community ecological and biogeographical concept. As such, it has profited from progress in community ecology punctuated by two major innovations: shifting focus from patterns to functions and changing approach from observational to explanatory (Fig. 6). This qualitative shift facilitates the ability to view a biome as a dynamic biological entity with many aspects, with deep roots in the evolutionary past and undergoing slow, yet profound multidirectional changes.

How do we understand the biome today? Let us summarize the points of consensus:

1. A biome is a large‐scale ecosystem occupying large spaces at least at the (sub)continental scale, or found in the form of a complex of small‐scale, isolated patches scattered across those large spaces.
2. A biome incorporates a complex of fine‐scale biotic communities; it has its characteristic flora and fauna and it is home to characteristic vegetation types and animal communities.
3. Biome patterns are driven by coarse‐scale (macroclimate) and meso‐scale (soil, water, disturbance) drivers, and the biome structures impose feedbacks on the environment.
4. A biome is generally characterized by a typical physiognomy (combination of plant and animal life forms), yet ecological feedback processes and disturbance may produce multiple stable states coexisting in the same geographic space.
5. A biome undergoes assembly (and disassembly) at both ecological and evolutionary timescales; the processes underpinning the assembly shape the functionality of the biome by selecting for the biota equipped by the best‐fitting set of traits matching the challenges of the environment.
6. Biomes can be modelled – predicted and retrodicted – at various levels of accuracy and precision.
7. Biomes are a useful ecological and evolutionary concept in terms of stratification of the biosphere into spatial and functional units.

In summary, a biome is a multiscale phenomenon, spanning several large‐scale spatial levels, including global climatic zones, continents and landscapes at subcontinental and supraregional scales. At all scales, they may show various vegetation‐physiognomic aspects that could represent multiple stable states. The patches of biomes are linked by a common network of ecological processes that define the selective pressures as macroclimatic, soil‐related, hydrological and natural large‐scale disturbance factors and stressors. A biome at any large spatial scale is a tangible, ecological‐evolutionary unit, carrying a legacy of deep evolutionary assembly processes. Depending on the extent of the spatial scale, we may be able to distinguish zonobiomes, subzonobiomes and continental/regional biomes.

Biomes are basic building blocks that make up the biosphere.
